# Harnessing smartphone technology and three dimensional printing to create a mobile rehabilitation system, mRehab: assessment of usability and consistency in measurement

**DOI:** 10.1186/s12984-019-0592-y

**Published:** 2019-10-29

**Authors:** Sutanuka Bhattacharjya, Matthew C. Stafford, Lora Anne Cavuoto, Zhuolin Yang, Chen Song, Heamchand Subryan, Wenyao Xu, Jeanne Langan

**Affiliations:** 1 Rehabilitation Science, University at Buffalo, Buffalo, NY USA; 2 Computer Science and Engineering, University at Buffalo, Buffalo, NY USA; 3 Industrial and Systems Engineering, University at Buffalo, Buffalo, NY USA; 4 Center for Inclusive Design and Environmental Access, University at Buffalo, Buffalo, NY USA

**Keywords:** Stroke, Rehabilitation, Smart technology, 3 dimensional printing, Measurement

## Abstract

**Background:**

Residual sensorimotor deficits are common following stroke. While it has been demonstrated that targeted practice can result in improvements in functional mobility years post stroke, there is little to support rehabilitation across the lifespan. The use of technology in home rehabilitation provides an avenue to better support self-management of recovery across the lifespan. We developed a novel mobile technology, capable of quantifying quality of movement with the purpose of providing feedback to augment rehabilitation and improve functional mobility. This mobile rehabilitation system, mRehab, consists of a smartphone embedded in three dimensional printed items representing functional objects found in the home. mRehab allows individuals with motor deficits to practice activities of daily living (ADLs) and receive feedback on their performance. The aim of this study was to assess the usability and consistency of measurement of the mRehab system.

**Methods:**

To assess usability of the mRehab system, four older adults and four individuals with stroke were recruited to use the system, and complete surveys to discuss their opinions on the user interface of the smartphone app and the design of the 3D printed items. To assess the consistency of measurement by the mRehab system, 12 young adults were recruited and performed mRehab ADLs in three lab sessions within 1 week. Young adults were chosen for their expected high level of consistency in motor performance.

**Results:**

Usability ratings from older adults and individuals with stroke led us to modify the design of the 3D printed items and improve the clarity of the mRehab app. The modified mRehab system was assessed for consistency of measurement and six ADLs resulted in coefficient of variation (CV) below 10%. This is a commonly used CV goal for consistency. Two ADLs ranged between 10 and 15% CV. Only two ADLs demonstrated high CV.

**Conclusions:**

mRehab is a client-centered technology designed for home rehabilitation that consistently measures performance. Development of the mRehab system provides a support for individuals working on recovering functional upper limb mobility that they can use across their lifespan.

## Introduction

The use of smartphones in healthcare has the potential to enhance the ability of individuals to self-manage health behaviors. Mobile apps have been used by individuals with diabetes to lower their blood glucose level more than a control group [1]. The use of mobile technology is also proving to be successful in increasing physical activity [2, 3]. With approximately 95% of Americans owning a mobile phone and 77% using smartphones [4], smartphones present an opportunity for accessible and adaptive apps in the management of health behaviors. While apps to improve management of diabetes or cardiovascular disease have been researched [5, 6], less is known about the use of smartphones to support maintenance or recovery of functional mobility in movement related disorders.

There is promise for the technology embedded in smartphones to be useful in the long term management of conditions such as stroke that result in sensorimotor deficits. Smartphones have been used to measure range of motion [7]. Furthermore, motion data collected by the inertial sensors in both Apple and Android smartphones, has been validated using a clinical motion capture system [8]. Touch screen technology can also capture timing of human movement for tasks such as tapping [9]. The smartphone represents portable technology capable of accurately quantifying movement and providing user feedback on motor performance through visual display and/or audio systems. To create a portable rehabilitation system, smartphones can be combined with three dimensional (3D) printed functional objects tailored to the individual’s mobility needs.

Creating automated systems that provide feedback on performance can promote participation, refine practice and give individuals a better understanding of their abilities to then set goals for themselves [10–12]. As demonstrated by previous research, exercise programs that include feedback from a person through a home visit, telephone call or clinic appointment have resulted in better outcomes compared to programs without feedback [10, 12]. With a robust body of research underscoring the importance of practice in improving motor abilities [13–16], providing adequate practice is central to creating efficacious interventions [10, 17].

The overarching goal of our research is to create technology that supports individuals with sensorimotor deficits self-manage upper limb rehabilitation at home. To this end, we created a portable rehabilitation system with the capacity of providing feedback on performance, mRehab (mobile Rehabilitation). Throughout the development of mRehab, we sought opinions from clinicians and individuals with stroke to refine the design. The design has progressed based on their responses [18, 19]. The aim of this study was twofold:

1. Assess the usability of the mRehab prototype based on feedback from older adults and adults with stroke and further refine the system based upon responses.
2. Using the updated system, examine if data from the smartphone embedded within a 3D printed functional item can be modeled to accurately count the repetitions of an mRehab activity, subsequently record the time taken to perform the activity, and consistently measure performance quality (smoothness or accuracy) across days.

Previous validations of smartphone hardware to evaluate movement [7–9] demonstrate smartphone technology is acceptable for recording human movement and allows us to focus on validating that our ADL modeling results in accurate counting of repetitions of mRehab ADLs and reliable measurements across days.

## Methodology

### Developing the early mRehab prototype

**Fig. 1:**
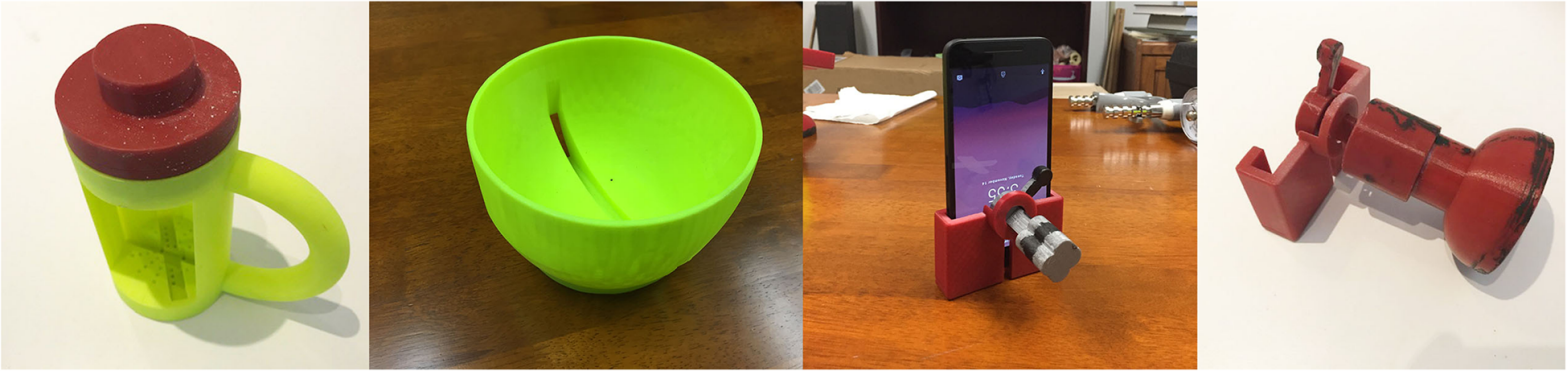
Prototype of the mRehab system – (from left to right) mug, bowl, key, doorknob

**Fig. 2:**
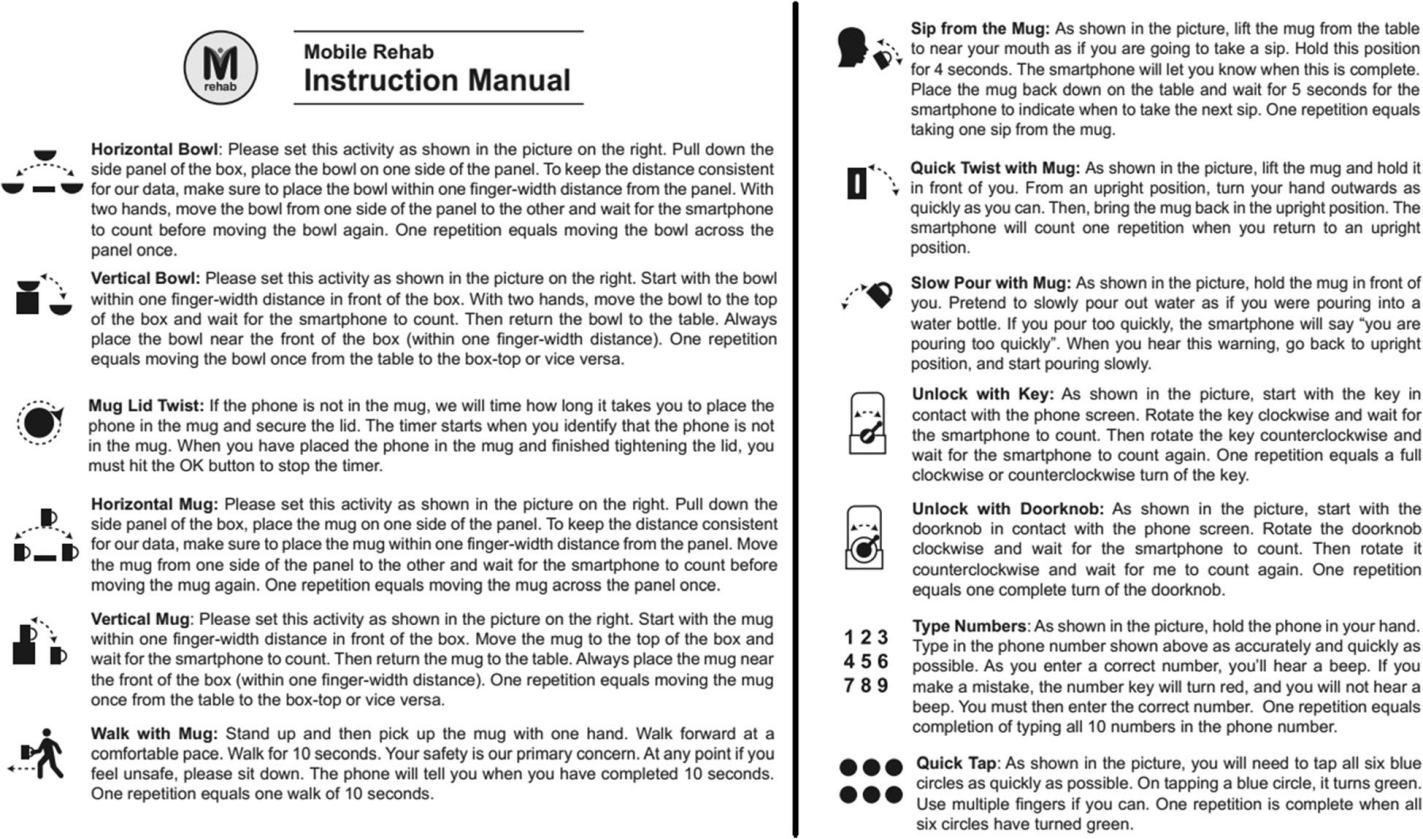
mRehab activities with description

**Fig. 3:**
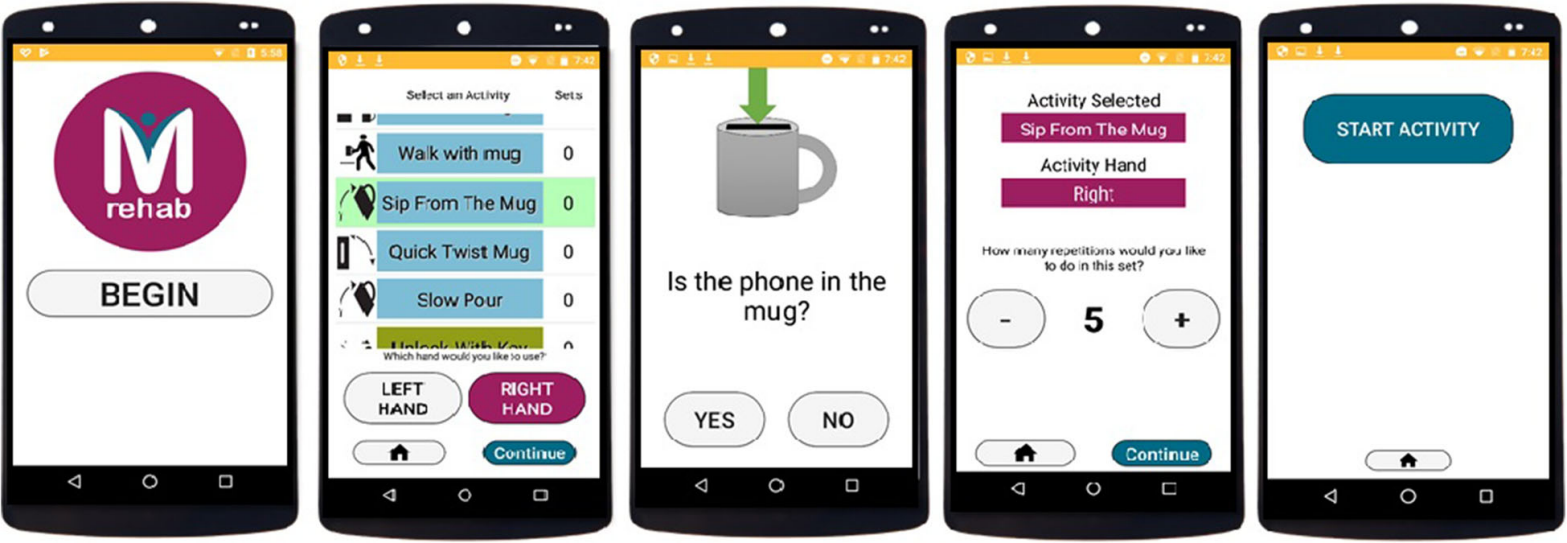
Step-by-step screen shots demonstrating the app interface

**Fig. 4:**
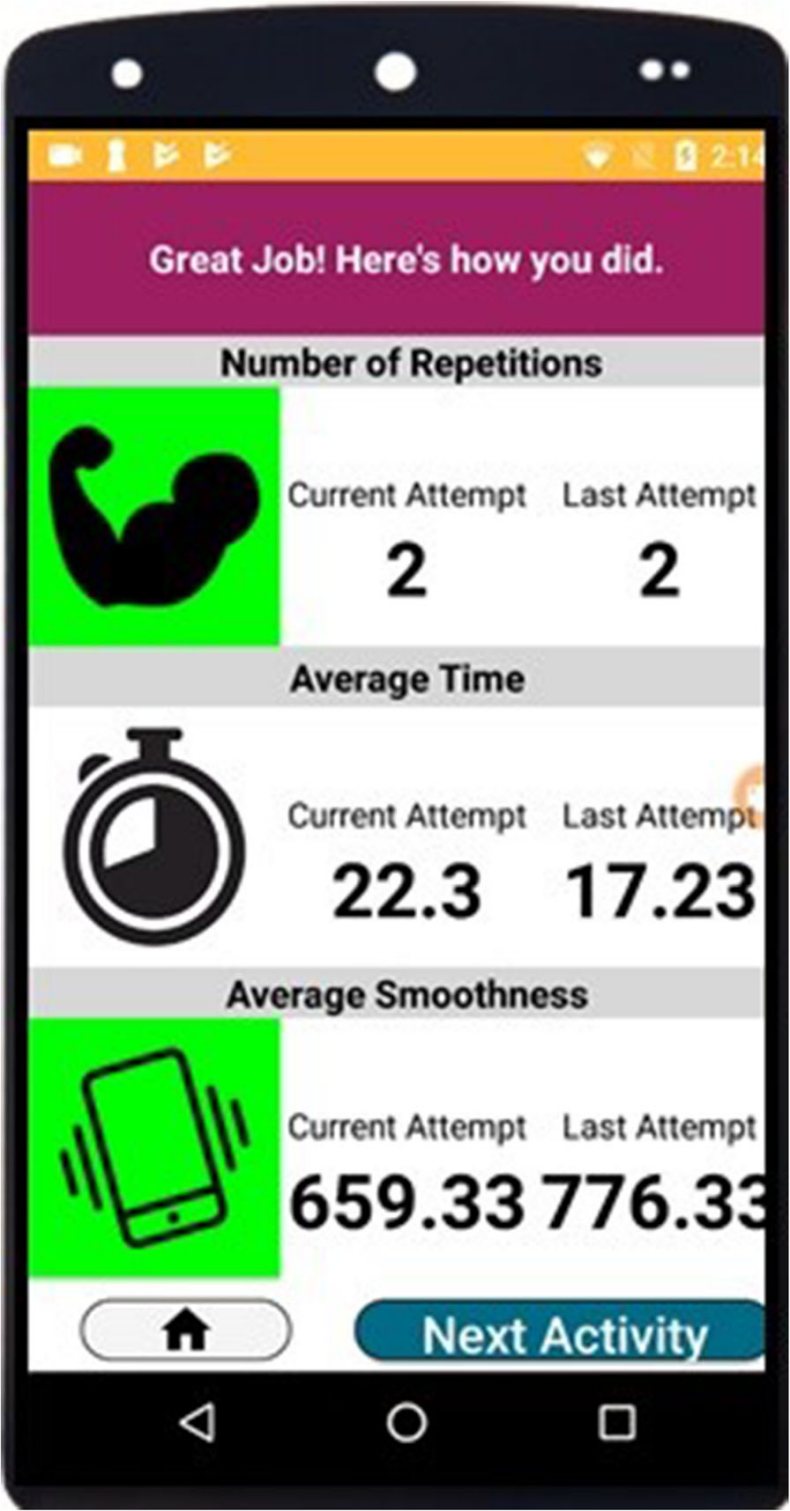
mRehab app ‘Feedback’ screen

We created a portable rehabilitation system with the capacity of providing feedback on performance, mRehab. The mRehab system consists of multiple 3D-printed functional items that, when combined with a smartphone, are used in a set of activities that mimic ADLs (see Table 1) with the smartphone app providing feedback on performance. The unique system includes 3D printed items that serve as household objects such as a mug, bowl, key, and a doorknob (see Fig. 1). The 3D printed items were designed to securely hold a smartphone (such as the mug or the bowl) or to hold the smartphone in place while the 3D printed lever swiped across the smartphone screen recording touch data capturing rotational movements for the key or doorknob. The Google Nexus 5 phone was used as the smartphone model for mRehab. A smartphone app was developed to record the data and present feedback on performance to the user. In this paper, we focused on development and refinement of the client interface. The client interface of the app allows the participant to select an activity from a list of 12 activities (see Table 1) which include unilateral and bilateral arm movements, elbow flexion and extension, forearm pronation and supination, and fine motor control (see Fig. 2). After the user selects an activity, they enter the number of repetitions for the set (Fig. 3). The app then provides verbal instructions to help guide the user through the activity. On completion of the set number of repetitions for the given activity, the smartphone app uses visual and auditory means to share performance scores based on the average for one repetition of the activity for duration, smoothness or accuracy of movement, and number of repetitions completed (see Fig. 4). The feedback screen also includes scores from previous performance in graph form, allowing the user to compare their current performance against their previous scores. Auditory and visual feedback emphasize when the score has improved compared to the previous session. When the participant’s score (number of repetitions, average time, average smoothness/accuracy) is better than the previous score, the specific icon turns green and (see Fig. 4) and also makes a celebratory auditory tone to notify the participant about their improved score.

**Table 1:** Activity descriptions, items used in the activity and associated smartphone assessments for the activity

Activity Name	Activity Instructions preceding each workout	3D item/app	Smartphone assessments
Vertical bowl	With two hands, move the bowl onto the top of the box and wait for me to count. Then return the bowl to the table and wait for me to count again.	Bowl with app	Repetition, duration, NJS
Horizontal bowl	Move the bowl with two hands from one side of the panel to the other. Set the bowl on the table and wait for me to count. Then, move the bowl again.	Bowl with app	Repetition, duration, NJS
Vertical mug	Move the mug onto the top of the box and wait for me to count. Then return the mug to the table and wait for me to count again.	Mug with app	Repetition, duration, NJS
Horizontal mug	You will move the mug from one side of the panel to the other. Set the mug on the table and wait for me to count. Then, move the mug again.	Mug with app	Repetition, duration, NJS
Sip from mug	Lift the mug from the table and bring it close to your mouth as if you are going to take a sip. Hold it there until I tell you to stop.	Mug with app	Repetition, duration, ZCR
Quick twist mug	Hold the mug in front of you in an upright position. As quickly as you can turn your hand outward and then return to the upright position.	Mug with app	Repetition, duration, ZCR
Slow pour	Hold the mug in front of you. Pretend to slowly pour-out water as if you are pouring into a water bottle. If you pour too quickly, I will say “You are pouring too quickly”. When you hear this warning, go back to upright position and start pouring slowly.	Mug with app	Repetition, duration, ZCR
Walk with mug	The app says “Stand up and pick up the mug with one hand. Walk forward at a comfortable pace till I tell you to stop”. During training, the user is explained that if they spill any (pretend) fluid from the mug, they will hear a spilling sound. They should straighten their mug and continue walking. The app times the user to walk for 10 s and then says “walk complete”, then gives a 5 s rest and then begins second repetition.	Mug with app	Repetition, duration, NJS
Unlock with key	With the key in contact with the phone screen, rotate the key clockwise and wait for me to count. Then rotate it counterclockwise, and wait for me to count again.	Key with app	Repetition, duration, ZCR
Turn door knob	With the door knob in contact with the phone screen, rotate the door knob clockwise and wait for me to count. Then rotate it counterclockwise, and wait for me to count again.	Door knob with app	Repetition, duration, ZCR
Phone number	Hold the phone in your hand. Type the phone number shown on the screen as quickly and accurately as possible.	App	Repetition, duration, accuracy
Quick tap	Tap all the blue circles as quickly as possible. Use multiple fingers if you can.	App	Repetition, duration, accuracy

### Assessing usability of the early mRehab system

**Fig. 5:**
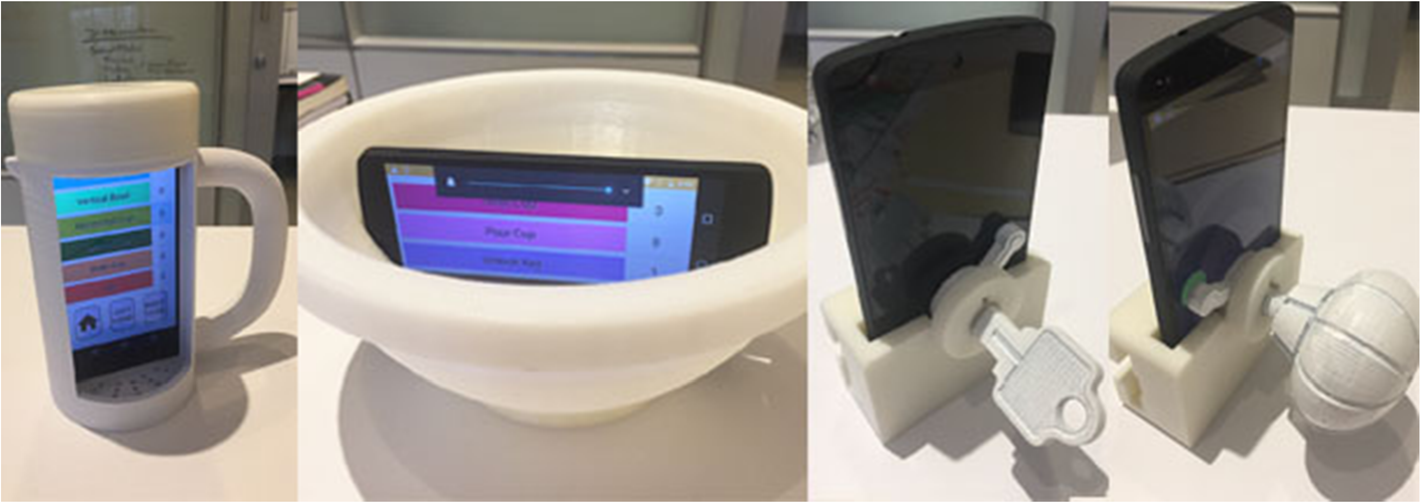
Revised prototype of the mRehab system (from left to right) mug, bowl, key, doorknob

Usability of the mRehab system was assessed by a convenience sample of four older adults (66–87 years, mean age = 72.5 years) and four individuals with stroke (54–68 years, mean age = 61.5 years). It was important to examine usability based on responses from older adults as this group may not be as familiar with technology as other age groups. Additionally, the risk of stroke roughly doubles every decade after the age of 55 [20]. Eight participants were identified to be an appropriate sample based on the 10 ± 2 participant rule for discovering 80% of the usability problems [21]. Three of the older adults and three of the individuals with stroke recruited in the study were females. All older adults were right handed. Two individuals with stroke presented with impaired right upper extremity and two presented with motor deficits of the left upper extremity. The study was approved by the University at Buffalo Institutional Review Board and all participants provided written informed consent for participation in the study. An occupational therapist instructed each participant on the use of the system, following which they engaged in the activities described in Table 1. The older adults were instructed to use their dominant arm, and the individuals with stroke were instructed to use their impaired arm to perform the activities. Following use of the mRehab system, participants provided usability ratings on a questionnaire modified from the Questionnaire for User Interaction Satisfaction [22] to answer a series of questions about their overall reaction to the system, system usability, and interface design. Researchers were available to provide the participants further clarifications on the questionnaire when necessary. The usability scale also included an open comments section for participants to address any additional remarks and explanations. The usability reports from the older adults and individuals with stroke were used to develop the updated mRehab system (Fig. 5) used in examining measurements of mRehab activities.

### Examining measurements of mRehab activities

**Fig. 6:**
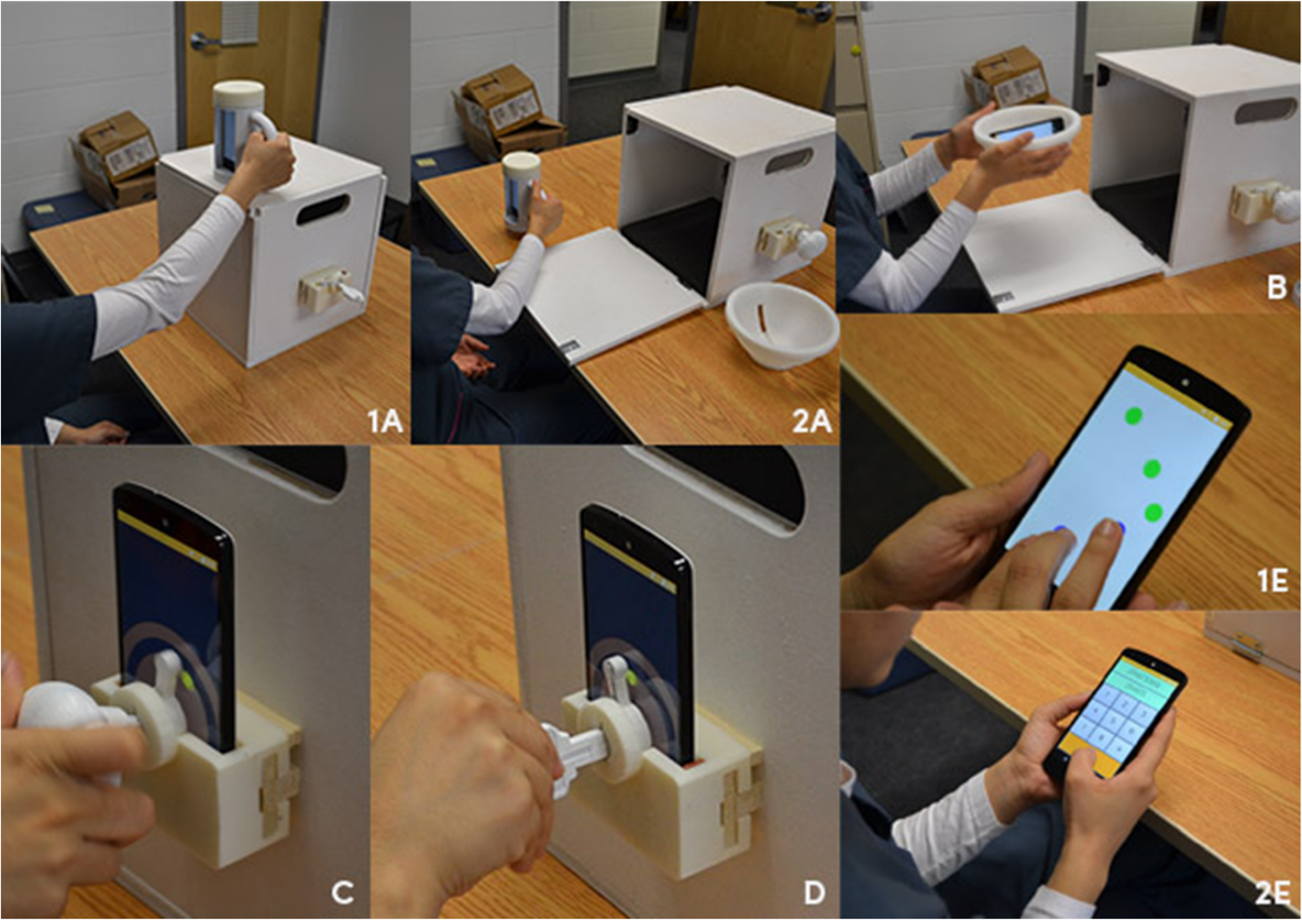
Activities in the mRehab system. **1a** and **2a**: Vertical and horizontal mug. **b** Horizontal bowl. **c** Turn doorknob. **d** Unlock with key. **1e** and **2e**: quick tap and phone number

The mRehab system was updated to more closely resemble household items and better guide the individual through the activities (further described in the results section). Figure 5 shows the updated 3D printed objects and the mRehab activities (Fig. 6) used in validating that our models of ADLs results in accurate counting of repetitions and reliable measurements across days. To assess the ability of the updated mRehab system to identify and record repetitions of an mRehab activity, concurrently recording data on time and smoothness of the repetition, and further examine consistency of measurement by the system across days, 15 young adults (18–30 years) were recruited using convenience sampling. The low variability in movement demonstrated by young, healthy adults makes this the preferred population to examine consistency of measurement [23–27]. Due to difficulties with scheduling, three participants missed their third visit, and therefore, were removed from the study analysis. Among the 12 participants included for the study, eight were female, all right handed, and ranged from 21 to 30 years of age (mean age = 24.67 years).

Participants visited the lab three times within 1 week. Two researchers facilitated the testing sessions at two different labs (seven participants were assessed in lab one and five in lab two). Based on findings from the usability study, a wooden 12”× 12”× 12″ box was used in this phase to standardize the distances for which the transfers were performed. Participants performed horizontal transfers across the 12” width of the box panel (see Fig. 5 - 2A and B), and vertical transfers from the tabletop to the top of the box (height of 12”) (see Fig. 5 – 1A). During the first visit, participants provided informed consent for participation in the study and consent to be video recorded. Then each participant engaged in three trials of a 10-s tapping task, using the index finger of their dominant hand to tap the “2” key on the numeric pad on a standard computer keyboard. The number of taps were recorded. This task was included as a control activity to examine consistency of motor performance with a device that participants commonly used, but was independent of the mRehab system. The researchers then demonstrated one of the activities shown in Table 1 and the participant immediately performed five repetitions of that activity using their dominant arm. This process was repeated until all activities were assessed. The mRehab system collected performance data on all activities listed in the manual except the “Mug Lid Twist” activity. This activity was included in the manual because it offers therapeutic practice; however, mRehab currently cannot measure the time taken to twist the lid on the mug. Therefore, Mug Lid Twist wasn’t a selection participants could select to practice on the list of activities on the graphic user interface (GUI).

During each activity, the app calculated the average performance in terms of smoothness of movement, zero-crossing rate, or accuracy depending on the activity (see Smartphone assessments for definitions), and the average duration of each activity. At the second and third testing sessions, the same procedure was followed. At the conclusion of the third visit, each participant received compensation for their time and effort.

Two forms of data were saved – raw sensor data (.csv) and calculated metrics (.json). The raw sensor data gave timestamps for each time the sensor updated and the acceleration values in the *x*, *y*, and *z* directions at that time. Both accelerometer and gyroscope data are saved during the rehabilitation process. The calculated metrics saved participant number, hand used, activity name, activity duration, activity smoothness/accuracy, activity repetitions completed, and date of completion. Copies of data are saved locally on the phone and backed up on the cloud with Amazon S3 server. The data security and privacy were ensured by a sophisticated infrastructure of network firewalls and strong access control. The current framework in Amazon S3 also supports implementation of another data encryption layer for database security if necessary in future studies. At this time participant data is used by participants to assist in self-management of their recovery and by researchers’ to examine changes in performance. It is not followed by therapists in a clinical setting.

Following the validity assessment of mRehab, two individuals with stroke used mRehab in their home for 6 weeks. The participants were instructed on how to use mRehab in the university lab by an occupational therapist. Participants were instructed to complete the mRehab activities using their impaired upper extremity. It was suggested that participants perform ten repetitions of each activity five times per week; however, it was explained that this is a participant centered program and participants may choose to perform lower or higher number of repetitions of an activity or sessions of exercise as they saw fit. During these 6 weeks, participants were encouraged to perform the Mug Lid Twist activity in addition to the other activities with their impaired arm at home, but there is no quantitative record. Both participants had caregivers who transferred the mRehab system from the university lab to the participant’s home and placed the system at a convenient location for the user. At the end of the 6 weeks, the participant’s compliance was examined through data collected from mRehab and ease of use of the mRehab system was assessed through an interview. We collected qualitative feedback from participants on the “Mug Lid Twist” activity.

### Smartphone assessments

Normalized jerk score (NJS) – Past literature indicates that NJS is an accepted way of measuring smoothness of movement [28–32]. NJS is computed as

$$ \mathrm{N} JS=\sqrt{\frac{1}{2}{\int}_{t_1}^{t_2}\left({\left({a}_x^{\prime}\right)}^2+{\left({a}_y^{\prime}\right)}^2+{\left({a}_z^{\prime}\right)}^2\right) dt\times \frac{\Delta  {t}^5}{a{m}^2}}, $$

where ${a}_x^{\prime },{a}_y^{\prime }, and\ {a}_z^{\prime }$ are the first derivative of the x-, y-, and z-axis accelerations (i.e., the jerk), *∆t* is the duration of a corresponding repetition (*∆t* = *t*_1_ − *t*_2_), and *am* is the amplitude of movement  [33]. In this case, the box was designed so that the *am* remained consistent for all movements. A NJS closer to zero implies smoother movement. A higher NJS results from greater changes in acceleration that accompany non-smooth or jerky motion. The app presented the average NJS for the five repetitions of the activities.

Zero-Crossing Rate (ZCR) – For activities involving quick rotation of the smartphone or across the screen of the smartphone, the NJS is insufficient due to the high number of changes in acceleration required for the activity. Therefore, the ZCR was adapted, based on a count of the number of times the signal (acceleration) switched from positive to negative (crossed zero) [34, 35]. ZCR was used for the Slow Pour, Sip from Mug, Quick Twist, Turn Key and Doorknob. For the key and doorknob, rather than capturing the rotational movement with the accelerometer, the smartphone touchscreen was used. A lever was devised to extend from these devices and make contact with the touchscreen. Conductive material was painted onto the lever and we were able to capture the x-y coordinate of the tip of the lever. This outermost tip relative to the pivot point gives us the angle of either the key or doorknob relative to the phone screen. Once we have the angle of the object we can capture the rotational velocity. The ZCR is applied to the values of the rotational velocity. As the key is turned in the opposite direction the velocity is reversed crossing over the velocity of 0 and adding to the total zero crossing count.

Accuracy – For the following activities: Phone Numbers and Quick Tap, accuracy rather than smoothness was relied upon. Each time the participant reached the appropriate endpoint for the activity, they were considered accurate. If they did not reach the designated endpoint, it was considered an error. On the feedback screen, the user viewed the average accuracy scores instead of the average NJS.

Duration – The duration calculated the time taken by the user to complete one repetition of each activity. On the feedback screen, the user was able to see the average duration it took for them to complete ‘n’ number of repetitions. The algorithms for duration differ based upon characteristics of the activity. For the Horizontal and Vertical Transfers with the bowl and mug, the object will rest on the table to start and return to the table at the end of the movement. Acceleration during a pickup will be one large positive spike, followed by the return to the table from the peak, a negative spike, and finally the placing on the table, another positive spike. Outside of these major spikes, there is also noise caused by trembles in the hand and imperfect path movement. The only point where the accelerometer has nearly no noise is when it has been placed back onto the table. To convert this into an algorithm we use two thresholds: major movement and minor movements. When the device is first picked up it crosses the major movement threshold setting the object into a state of ‘moving’. Once moving, the code looks for the point at which the object is again at rest. This is determined by a lower threshold, the minor movement. The code identifies when the acceleration is within the bounds of the minor movement threshold and that is considered the end of the repetition. We then set the state to ‘not moving’ and again look for a major movement.

For the key and doorknob activities, we set the angle the object needs to be rotated to count as a repetition. We achieve this with the conductive point at the end of the lever. Since we know the origin of the lever’s pivot point and the radius of the arm, we are able to calculate how far the lever has been turned. For the sip from the cup activity, once the participant rotates past the required angle to trigger the activity, it requires that the participant hold that position until all liquid is gone. The phone number and quick touch both use levels, once the phone number is correctly entered or the dots are all touched, the repetition is over. Lastly, the twist cup activity determines the end of the repetition by using the accelerometer. A typical turn consists of a rotation outwards pointing the top of the mug towards the side and a rotation inwards pointing the top of the mug to the sky. This movement results in a sharp negative acceleration followed by a sharp positive acceleration. We have a threshold set that if broken first by a negative value then followed by a positive value we consider this a completed repetition.

### Measures and statistical analysis

The usability reports were descriptively analyzed. In order to assess the consistency in the system measurements, the average duration and smoothness of movement were recorded for each activity on each day and used in subsequent analysis. The ability of mRehab to identify and record repetitions was verified by the researcher observing the participant and listening for the audio count during the activity followed by looking at the screen for the repetition count at the completion of the activity. The testing sessions were videotaped, if there was a need for review.

Absolute reliability [34] of measurements by mRehab for duration and smoothness measured across the replicated tests was evaluated by the coefficient of variation (CV = standard deviation/mean × 100). Variability less than 10% of the mean (CV < 10%) was set as the target for good reliability [36–39]. Because we expect a certain level of variation in human performance [27] using CV for statistical analyses rather than interclass correlation coefficient (ICC) is appropriate. ICC is commonly used to assess variability between trials within a day, its interpretation can be influenced by inconsistency in the direction of the variability between trials (e.g., a learning effect vs. random variation). We believed that healthy young adults would perform the mRehab activities with proficiency and would not demonstrate motor learning with the activities, only random variability. All statistical analyses were conducted in SPSS v. 24 (IBM Corporation). Two measures were excluded from analysis of variation: duration of the walking activity and accuracy of the phone number activity. For the walking activity, the duration was controlled at 10 s, and for the phone number task all participants had 100% accuracy for all trials.

## Results

### Assessing usability of the mRehab system

The findings from the usability scale with the older adults and individuals with stroke provided information about the usability of the system. Quantitative usability ratings from participants are summarized in Table 2. When given the usability scale, participants requested further clarifications regarding the ‘level of progress’ on the usability scale. The researchers explained that it referred to the performance feedback screen and the auditory and visual cues on the phone – “Was the feedback from this screen sufficient for them to understand if they had made progress with the activity?” No other questions on the usability scale required clarification. Overall, participants positively rated the usability of the mRehab system. Participants commented that the activities were “interesting” and “similar to everyday tasks” unlike working with “inanimate objects like clay and elastic strap”. Clarifying with the participant, “clay” referred to theraputty and “elastic strap” referred to theraband. These items are commonly provided with written home exercise programs [40]. Participants provided suggestions for improvements to mRehab, which included – decreasing the number of turns needed to open and close the screw-top lid of the mug, modifying the design of the key and doorknob to more closely resemble real life objects, and increasing the clarity and consistency of instructions across ADLs for the app. They further advised to refine the system by using more consistent language for the activity instructions. Participants appreciated the portable nature of the rehabilitation system, and identified some positive features which included relevance of activities to daily life, ease of learnability and overall ease of use of the system.

**Table 2:** Usability ratings from older adults (*n* = 4) and individuals with stroke (*n* = 4)

Usability items (on a scale of 9)	Mean	Standard Deviation
Overall reaction - frustration to satisfying	6.6	1.85
Overall reaction - dull to stimulating	6.50	3.12
Ease of reading characters on the screen	7.88	2.10
Clarity on the level of progress	7.43	1.72
Ease of learning to operate the system	8.75	0.46
Clarity of the sequence of screens	8.63	0.74
Clarity of the organization of information	8.5	0.76
Clarity of activity performance	7.57	1.62

Based on the findings, the updated prototype of mRehab was built (Fig. 5). The handle of the mug was modified to a more preferred ‘D’ shape and the number of twists needed to open and close of the mug lid were reduced. Both right and left handed mugs were created so that the phone screen is visible to participants regardless of their affected side. A lip was added to the edge of the bowl to prevent it from accidentally slipping from the hands of the participants. Moreover, the design of the bowl was modified to be more rounded to facilitate supination of forearms. Design for key and door knob was also revised to more closely resemble real life shape and size. The color of the 3D printed items were changed to a neutral white color, a box was built to standardize distances objects were moved in the activities and contain all study items. The instructions on the app were revised to provide more clarity and consistency across activities. To increase clarity, in addition to audio instructions on the app, one page manuals were created with written instructions for the activities.

### Examining measurements of mRehab activities

The app was able to successfully recognize 100% of the repetitions correctly, validating our algorithms for each of the mRehab activities. The assessment of consistency of measuring human movement resulted in a range for CV for the mRehab tasks. The low end of this range neared the CV for the control task, tapping on the computer keyboard. The CV for the computer keyboard tapping task over the 3 days was 6.2 (± 3.8%). Table 3 reports performance on duration of activities in mRehab. For activity performance duration, variability about the mean across the replications ranged from 6.3% (for sip from mug) to 46.2% (for quick twist mug). Table 4 reports performance on smoothness for activities in mRehab. For smoothness, CV ranged from 7.1% (for slow pour) to 53.3% (for quick twist mug). Overall, the quick twist mug activity had the highest variability in measurement across days. The lowest variability for both metrics was observed for the slow pour activity. Generally, higher variability was observed for smoothness compared to duration.

**Table 3:** Coefficient of variation for average duration of performance

Activity Name*	Average duration in seconds (Standard Deviation)			% Coefficient of Variation (SD)
	Day 1	Day 2	Day 3	
Vertical bowl	4.3 (0.29)	4.6 (1.0)	4.2 (0.4)	9.2 (8.9)
Horizontal bowl	4.4 (0.7)	4.2 (0.5)	4.3 (0.7)	11.1 (7.5)
Vertical mug	4.2 (0.55)	4.2 (0.41)	4.1 (0.47)	7.7 (5.3)
Horizontal mug	4.5 (0.85)	4.1 (0.36)	4.4 (0.53)	9.9 (7.3)
Sip from mug	10.0 (0.95)	10.2 (0.86)	10.2 (0.84)	6.3 (5.2)
Quick twist mug	1.4 (1.1)	1.6 (1.1)	2.3 (2.6)	46.2 (31.4)
Slow pour	19.8 (2.6)	19.4 (1.1)	19.1 (1.0)	6.8 (4.9)
Phone number	9.4 (1.3)	9.3 (1.5)	8.8 (0.9)	9.0 (6.0)
Quick tap	5.4 (1.3)	6.2 (0.6)	6.0 (0.7)	10.8 (13.5)

*Note: Walk with the mug is not included in this table, as the duration of 1 repetition was fixed to be 10 s

**Table 4:** Coefficient of variation for average smoothness/accuracy of performance

Activity Name*	Average smoothness/accuracy (Standard Deviation)			% Coefficient of Variation (SD)
	Day 1	Day 2	Day 3	
Vertical bowl	394.2 (52.9)	376.7 (52.1)	357.5 (72.0)	13.0 (10.3)
Horizontal bowl	372.0 (231.8)	332.5 (78.5)	452.1 (280.4)	22.1 (19.6)
Vertical mug	357.8 (61.5)	373.0 (69.1)	361.1 (56.9)	14.5 (7.5)
Horizontal mug	375.5 (73.9)	368.2 (61.1)	398.8 (82.1)	13.8 (7.8)
Sip from mug	378.7 (88.2)	392.4 (63.7)	393.1 (80.5)	12.5 (10.2)
Quick twist mug	155.3 (121.6)	190.8 (152.3)	296.4 (374.5)	53.3 (34.3)
Slow pour	648.9 (82.5)	625.6 (41.2)	622.4 (39.6)	7.1 (4.8)
Walk with mug	1046.0 (127.2)	1063.4 (120.9)	1071.9 (128.4)	8.2 (3.8)
Quick tap	−2.2 (105)	1.1 (95.5)	22.1 (64.4)	−2.6 (121.2)

*Note: Phone number is not included here because all participants had 100% accuracy with the phone number task making the CV = 0

The design of the key and the doorknob did not hold up to repetitive use when assessing consistency of measurement. For both key and doorknob activities, the 3D printed object broke during testing, thereby reducing the sample size to seven participants who completed these activities in all three testing sessions. Due to this, the consistency of measurement with performance with the key and the door knob was dropped from the current analysis. The defect in the physical design was corrected in preparation for future pilot testing (described in the discussion section).

Individuals with stroke who used mRehab in their home for 6 weeks reported that they left the system in one convenient location. They reported that they independently set-up the system which took less than 5 minutes prior to initiating activities using mRehab. They also commented that the GUI of the app was simple and easy to use since it offered step-by-step instructions. The participants used the system 16 and 29 days respectively, completing all of the activities guided by the smartphone app. Participants reported that they occasionally forgot to perform the Mug Lid Twist activity with their impaired arm because the app did not include it as a separate activity and no feedback was available for this activity.

## Discussion

A survey of physical and occupational therapists found that 87% send patients home with written home programs. Furthermore, 74% of the therapists reported including non-technological equipment such as theraputty or theraband in their clients’ home programs. However, relatively few therapists reported including technology in their clients’ home programs [40]. Adherence to written home programs is not strong [12, 41, 42]. Patients have reported lack of instructions and motivation contributing to their dismissal of home programs [41]. Use of mobile technology in rehabilitation can provide the opportunity to more fully engage participants in their recovery promoting adherence with home rehabilitation. Participants in this study indicated that they prefer the mRehab activities over “clay” and “elastic strap” because the activities resembled daily tasks. The ability of the participants to foresee these activities in the context of their day-to-day life can be beneficial to overcome perceived barriers when problem-solving upper extremity use at home [43]. Task-specific training has been shown to be effective in rehabilitation [44, 45]. Common objects instrumented with a miniature motion tracking sensor for task-specific training (grasping and moving the object) with feedback on performance parameters, such as speed and accuracy, have resulted in improvements in dexterity for a small group of participants with sensorimotor deficits of the hand [46]. Patient’s reported this training encouraged them to be competitive and made the experience enjoyable [46]. However, the system used for this training was lab-based and not applicable to home programs. Fully engaging users with technology and meaningful activities can lead to better outcomes.

Participants’ overall reaction to the mRehab system was generally positive, except for one older adult and one individual with stroke. The older adult found the activities to be very easy to perform and therefore, did not find them to be stimulating (scored 0 on a scale of 9). On the other hand, the activities with the key and doorknob were too difficult to perform for one individual with stroke who found the activities to be less satisfying (score of 3 on 9). These participant responses suggest that finding the “just right” challenge for each individual in their home program is very important. High usability ratings by participants on clarity of level of progress (mean = 7.43, SD = 1.72) and clarity of activity performance (mean = 7.57, SD = 1.62) indicated satisfaction with the performance feedback received from the app. The clarifying comments participants made regarding the consistency of language in the activity allowed us to take a satisfactory feature and further refine it. The usability survey responses with older adults and individuals with stroke provided valuable information that was used to modify the design of mRehab.

mRehab promotes a client centered approach. The use of 3D printing technology allows customization of the items used in training activities. We received positive comments from participants on the use of 3D items in functional activities. Participants could see how practicing these activities was relevant to improving their functional mobility. The use of 3D printing allows tailoring of the 3D functional items to meet the needs of the client, such as customizing the mug handle, shape of the bowl, size of the key or the door knob – the possibilities are extensive. The app also allows customization, enabling the participant to make choices in the activities they perform and on the number of repetitions they perform. They can choose to concentrate their efforts on the activities they find most meaningful, because engaging in meaningful task-specific training can result in significant upper extremity recovery for individuals with stroke [44].

We examined the consistency of feedback when young, healthy adults performed the activities in mRehab. Our target of having a CV less than 10% is in line with previous literature [38, 39]. We further feel it is justified in that the control activity, computer keyboard tapping resulted in a CV of less than 10%. Our goal of having a CV that was less than 10% was met for six activities when measuring duration of performance, including – Vertical Bowl, Vertical Mug, Horizontal Mug, Sip from Mug, Slow Pour and Phone Number. When measuring smoothness of performance, the coefficient of variation was less than 10% for four activities (Slow Pour, Walk with Mug, Phone Number and Quick Tap). Several activities were near this target with a CV of less than 15%. This included: Horizontal Bowl and Quick Tap for performance duration and Vertical Bowl, Vertical Mug, Horizontal Mug, and Sip from Mug for smoothness and accuracy. Smoothness for Horizontal Bowl and both duration and smoothness for Quick Twist mug had CVs above 20%. On further analysis, we noted that the smoothness of the Horizontal Bowl activity was exceptionally high (more than 2× the average) during the third trial for two participants. The high smoothness scores of these two participants resulted in 57 and 67% within subject CVs, and thus a high mean CV. Because this activity targeted moving quickly and had less of an ADL application, it is possible that their performance is less stable than performance on the other activities. The CV for average duration and smoothness for the Quick Twist activity was 46 and 53% - therefore this activity is no longer a standard part of the program. The large CV for this activity makes it undesirable in a home program that tracks performance. The day to day variability is so high that it would be challenging for the client to determine if they are actually improving and could lead to frustration. With the majority of activities resulting in CV for duration and smoothness near or below our target of 10%, it demonstrates smartphone technology is a viable method of collecting movement data in home rehabilitation programs.

When examining measurements of the mRehab activities, we found that parts of the key and doorknob broke under stress of repetitive use due to minimal infill. We changed the infill density (filament printed inside the part) of each print to 100% to increase the part strength. We also modified the print orientation by changing the direction of the filament structure to increase tensile strength. An additional layer of epoxy coating was added to strengthen the products and protect the conductive paint from wearing off prior to in-home evaluation to improve durability to repeated use. The added weight provided a better design because the objects felt more realistic to the user.

Individuals with stroke used the system in their home. They appreciated the simplicity of the app GUI and felt comfortable with its use. Although the manual included the “Mug lid twist” activity and the participants were verbally instructed to use the mug screw-top with their impaired side, they reported performing this activity inconsistently due to lack of reminders. This emphasizes the need for reminders about activity performance and the significance of providing objective feedback.

This study assessed early prototypes of the mRehab system which prepares us for the next phase, a pilot study. Testing early prototypes is necessary to evaluate the usability and reliability of a product which provides the opportunity to improve the design, but the testing was not exhaustive. We did not control for any external factors that could influence the participant’s performance, such as time of the day, or fatigue. It is possible that controlling for these factors would have resulted in better CV for the activities. Data collection was conducted at two different sites. We found similar results from both sites for nearly all the activities. Differences in duration and smoothness/accuracy scores were only significant between sites (using an independent samples t-test) for 1 day for Horizontal Bowl smoothness and 2 days for Horizontal Mug smoothness (at the *p* < 0.05 level). All other differences were not significant, indicating consistent measurement independent of testing location, which adds to the strength of the mRehab system as a reliable tool for measurement in different home environments.

This study has furthered the development of mRehab in preparation for a pilot study. There are limitations to this research. At this point of development, we have only considered, but not tested the use of mRehab with other smartphones including a participant’s personal smartphone. Three dimensional printing allows for physical changes in the 3D objects to accommodate varying sizes of smartphones. The mRehab app utilizes hardware components such as accelerometer, gyroscope, touch screen and CPU + RAM that are readily available in all smartphones. We do not anticipate that hardware updates in future smartphones will affect the function of mRehab. Another aspect to address will be the ability to use one’s personal smartphone for mRehab and the potential for an incoming call. Depending on the user’s request, any incoming calls could be disabled when the mRehab app is running. Most smartphone systems, including iOS and Android support this function, such as entering “Do Not Disturb” mode while using mRehab. We will also need to examine the amount of time it takes participants to set up the system. We hypothesize that the duration and difficulty level would greatly vary based on the individuals’ abilities and their environmental context. Participant might choose to initially setup the system and leave it as is for future use; or they might set up and take down the system for each session. Nonetheless, future usability testing will assess time and difficulty of self-setup. These questions will be addressed in further development of the system and future research. Additionally, we plan to develop a therapist-interface in the future iterations of the app so that the performance scores from the mRehab-users can reach their corresponding therapists.

Use of mobile technology is on the rise globally. Individuals with disabilities in rural and remote areas in low and middle income countries often have poor access to healthcare professionals. The decreasing costs and increasing network coverage are enabling mobile phones to become a common commodity in low and middle income countries with subscription rates as high as six billion [47]. The portable mRehab system has the potential to support rehabilitation in remote areas of the United States and globally.

## Conclusion

With robust evidence demonstrating that improvements can still be made several years post-stroke [48, 49]. Developing approaches to better support clients with their home program is important. Use of mRehab is a promising avenue for promoting self-management of rehabilitation across their lifespan. With rise in use of mobile phones within the United States [4], and globally across the world [47], novel utilization of mobile technology will help to develop inexpensive tools for rehabilitation. The mRehab system uses an innovative approach to couple 3D printing technology with smartphones that has the potential to revolutionize use of technology in home programs and enable provision of affordable home programs to individuals in all parts of society.

## Data Availability

The datasets used and/or analyzed during the current study are available from the corresponding author on reasonable request.

## Abbreviations

3D: 3 dimensional
ADL: Activities of daily living
CV: Coefficient of variation
ICC: Interclass correlation coefficient
mRehab: mobile rehabilitation
NJS: Normalized jerk score
ZCR: Zero-Crossing Rate
